# Health Information Avoidance and eHealth Literacy in Patients With Multiple Sclerosis: A Cross‐Sectional Correlational Study

**DOI:** 10.1002/hsr2.71418

**Published:** 2025-10-28

**Authors:** Leila Mobasher, Maryam Shekofteh, Maryam Kazerani, Sara Jambarsang, Maryam Poursadeghfard

**Affiliations:** ^1^ Department of Medical Library and Information Science, School of Allied Medical Sciences Shahid Beheshti University of Medical Sciences Tehran Iran; ^2^ Center for Healthcare Data Modeling, Departments of Biostatistics and Epidemiology, School of Public Health Shahid Sadoughi University of Medical Sciences Yazd Iran; ^3^ Clinical Neurology Research Center Shiraz University of Medical Sciences Shiraz Iran

**Keywords:** electronic health literacy, health information avoidance, multiple sclerosis

## Abstract

**Background and Aims:**

Information avoidance refers to deliberately evading or delaying access to freely accessible but undesired information, and eHealth literacy refers to people's capability to comprehend, assess, find, and use health knowledge derived from electronic sources. The present study aims to investigate the health information avoidance and eHealth literacy levels in patients with multiple sclerosis (MS) in Fars Province, Iran, and their correlation with each other.

**Methods:**

The present research method was a cross‐sectional correlational study. The studied population included all patients with MS in Fars Province, Iran, in 2023. The sampling method was convenience sampling with a sample size of 127 people. The data collection tools consist of the Health Information Avoidance Questionnaire and the eHEALS Questionnaire. Data analysis was performed using descriptive statistics, Pearson's correlation coefficient, *t*‐test, ANOVA, and Bonferroni test with the help of the SPSS software version 21.

**Results:**

The average score for general health information avoidance was 9.06 out of 20 points, while the average score for MS health information avoidance was 20.52 out of 50, and the average patient eHealth literacy score was 26.28 out of 40 points. However, this analysis found no significant correlation between general health information or MS health information avoidance and eHealth literacy avoidance (*p* ≥ 0.05).

**Conclusion:**

Most patients show an above‐average understanding of health information literacy and minimal health information avoidance. Nevertheless, micro‐and macro‐level strategies and policies are necessary to decrease this avoidance and enhance eHealth literacy optimally. However, because of the convenience sampling, the generalization to the broader population should be approached with caution.

## 1 Introduction

The interaction between humans and information holds a significant role in information science research. Information behavior includes searching for information, as well as all non‐intentional, passive, or purposeful behaviors related to it [1]. Information avoidance is a type of information behavior in which a person actively or passively avoids sources and information that lead to a negative feeling or result [2]. Information avoidance represents a motivated decision to avoid information threatening one's thoughts, feelings, or behaviors [3]. People's readiness to accept different types of information is different, and they may exhibit a greater tendency to avoid acquiring certain types of information [4].

In the health area, the use and promotion of health information can effectively empower people to prevent, control, and manage diseases and make medical decision‐making. It also promotes self‐care through health information‐seeking behaviors [5]. Health information enhances the community's knowledge of health and the quality of life. Consequently, in dealing with diseases, health information helps patients feel less anxious [6]. However, despite the positive consequences of health information, some people prefer to avoid the information [1, 7, 8, 9]. Consequently, information avoidance may potentially lead to adverse consequences for social engagement, the relationship between patient and healthcare provider, and overall health [10]. More health information avoidance is consistently related to beliefs, indicating lower health agency [11].

Multiple sclerosis (MS) is an autoimmune disease that targets the central nervous system, explicitly damaging the myelin sheath of brain and spinal cord cells. This often leads to varying degrees of disability, posing significant burdens on the health system and the patients. Alarmingly, the prevalence of MS is consistently rising [12]. This disease is primarily diagnosed in the age range of 20−40 years, and its prevalence is two to three times higher in women than in men. In addition to affecting various aspects of patients' lives, this disease brings a significant cost and burden to the health system [13].

Several studies have been conducted on the topic of information avoidance. For instance, Cavlovic et al. developed a model which predicted increased information accuracy leads to decreased information avoidance [14]. Naderbeigi and Isfandyari‐Moghaddam's research indicated that individuals suffering from reluctance to access information deliberately limit their search for and use of information. Consequently, they miss opportunities to acquire new information and often rely on a few limited sources of information [15]. In another study, Naderbeigi and Isfandyari‐Moghaddam found the average information avoidance in medical sciences students to be 2.21 out of 5 [16]. Schumacher et al. demonstrated that the average value of information avoidance related to weight exhibited a low magnitude, precisely 2.23, less than half of the total [17]. Howell and Shepperd showed various levels of information avoidance in different groups. The average avoidance score for attractiveness information was 4.05, while information related to health was 2.44, and information about partner infidelity was 1.87 [8].

To plan to reduce health information avoidance, it is necessary to consider the factors affecting it. In this regard, the research of St. Jean et al. showed that people with less health literacy tend to have more information avoidance [4]. Siebenhaar et al. investigated the relationship between electronic health literacy (eHealth literacy) and health information avoidance. His findings showed that people with a higher level of eHealth literacy experienced less worry and anxiety during the COVID‐19 pandemic and, as a result, had less information avoidance [18]. Therefore, eHealth literacy is one of the factors affecting information‐seeking behavior and health information avoidance [18]. eHealth literacy refers to people's capability to comprehend, assess, find, and use health knowledge derived from electronic sources. Its goal is to enable individuals to make informed healthcare decisions, enhance health outcomes, and diminish health disparities [9]. However, more evidence is needed regarding the relationship between eHealth literacy and health information avoidance. A literature review shows that no studies have been conducted to examine information avoidance or eHealth literacy in patients with MS. Accordingly, the present study aims to investigate the status of health information avoidance in patients with MS and its relationship with the eHealth literacy level. The present research findings, while showing a clear situation of information avoidance behavior and eHealth literacy in the research population, can be used by health information administrators to plan and make necessary policies to create the required platforms for information‐seeking and promoting eHealth literacy.

## 2 Methodology

### 2.1 Study Design, Population, and Samples

In this study, conducted by a cross‐sectional correlational method, the investigated population includes patients with MS in Fars Province, Iran, in 2023 (more than 4000 people). The sample size was calculated by considering the initial correlation value at a 0.15 level, type 1 error at a 0.05 level, and 80% power, 127 samples. Nevertheless, we distributed and collected 130 questionnaires among patients. The sampling was a convenience method, completed by visiting Imam Reza Clinic and Shahid Faghihi Hospital, Shiraz, Iran (Brain and Nerves Ward). All participants were between 14 and 71 years old and provided written informed consent for their participation in the study. Notably, the inclusion criterion for the study was the patients' consent to participate. Since some participants were under 18, we also obtained written informed consent from their parents.

The questionnaires were distributed and collected in person and the data collection period for this study was one and a half months, starting on March 30, 2023, and ending on May 15, 2023.

### 2.2 Data Collection Tool

The data collection tools include three sections: (1) Demographic information; (2) The Health Information Avoidance Questionnaire; (3) The Electronic Health Literacy Questionnaire.

#### 2.2.1 The Health Information Avoidance Questionnaire

This questionnaire is designed based on Howell and Shepperd's Health Information Avoidance Questionnaire, which has been used in people over 18 years of age [8]. The population of the present study was MS patients whose ages ranged from 14 to 71 years. To tailor the questions to the age groups between 14 and 18 years, we tried to design the questions as simple and understandable as possible and not to use long sentences or specialized terms. The research team confirmed the face validity of the questionnaire, and the internal reliability was confirmed using Cronbach's *α* for 25 people from the study population (0.74). In addition, one of the researchers distributed the questionnaires in person and provided the necessary explanations to the respondents in case of ambiguity.

The questionnaire is divided into two parts: (I) General health information avoidance and (II) MS health information avoidance.

(I) General health information avoidance section: The researchers added two questions to Howell and Shepperd's general health information avoidance section: Question 3, “I usually do not want to be exposed to information that contradicts my previous knowledge or belief,” and Question 4, “Fear of wrong information makes me not look for information about the disease.” Responses to these questions were set on a 5‐point Likert scale ranging from *strongly disagree* (score 1) to *strongly agree* (score 5), and question 2 was scored inversely. The score range of this section was between 4 and 20. The questions in this section are the same items shown in Table 1.

**Table 1:** The frequency of patients with MS in terms of general health information avoidance.

Items	Strongly agree F (%)	Agree F (%)	Neither agree nor disagree F (%)	Disagree F (%)	Strongly disagree F (%)
In general, I would avoid learning health information about myself	4 (3.1)	11 (8.5)	15 (11.5)	47 (36.2)	53 (40.7)
Even if it upsets me, I generally want to know health information about myself	56 (43.1)	53 (40.7)	11 (8.5)	6 (4.6)	4 (3.1)
I usually do not want to be exposed to information that contradicts my previous knowledge or belief	12 (9.2)	12 (9.2)	42 (32.3)	43 (33.1)	21 (16.2)
Fear of wrong information makes me not look for information about the disease	15 (11.5)	17 (13.2)	27 (20.8)	45 (34.6)	26 (19.9)

*Note:* (R) means that this item was scored reversely.

(II) MS health information avoidance section: This section is a researcher‐developed questionnaire designed based on the 8‐item questionnaire of Howell and Shepperd. Howell and Shepperd's questions contain the blank parts to complete regarding the different domains [8]. Considering MS disease, we filled in the blank parts. For example, the item “I want to know _______” was completed to “I want to know the status of my MS (disease progression, new treatment methods).” Besides, the number of questions was increased to 10, such as Price [19]. The first item was “I would rather not know___.” Two questions were designed for this item (items 1 and 2 in Table 2). The other item was “It is important to know _____.” The researchers designed two questions for this item (items 8 and 9 in Table 2).

**Table 2:** The frequency of patients with MS in terms of MS health information avoidance.

	Items	Strongly agree	Agree	Neither agree nor disagree	Disagree	Strongly disagree
1	I would rather not know the information about the progression of my MS disease	17 (13.1)	9 (6.9)	11 (8.5)	48 (36.9)	45 (34.6)
2	I would rather not know the new treatment methods for my disease	3 (2.3)	4 (3.1)	12 (9.2)	58 (44.6)	53 (40.8)
3	I would avoid learning information about my MS (e.g., the rate of disease progression, new treatment methods)	2 (1.5)	10 (7.7)	17 (13.1)	48 (36.9)	53 (40.8)
4	Even if it upsets me, I want to know information about the status of my disease (disease progression, new treatment methods) (R)	57 (43.8)	49 (37.7)	14 (10.7)	10 (7.8)	0 (0)
5	When it comes to information about my MS status (disease progression, treatment methods), [sometimes] ignorance is bliss	15 (11.5)	20 (15.4)	36 (27.7)	35 (26.9)	24 (18.5)
6	I want to know the status of my MS (disease progression, new treatment methods) (R)	57 (43.8)	57 (43.8)	10 (7.8)	6 (4.6)	0 (0)
7	I can think of situations in which I would rather not know information about my illness (situations such as being with friends, family, etc.)	28 (21.5)	47 (36.2)	32 (24.6)	11 (8.5)	12 (9.2)
8	It is important to know the information about the progression of my MS disease (R)	69 (53.1)	41 (31.5)	17 (13.1)	3 (2.3)	0 (0)
9	It is important to know the control and new treatment methods for MS disease (R)	79 (60.7)	40 (30.7)	10 (7.8)	1 (0.8)	0 (0)
10	I want to know the information about the progression of my MS immediately after the MRI (R)	79 (60.7)	33 (25.4)	14 (10.8)	4 (3.1)	0 (0)

*Note:* (R) means that these items were scored reversely.

Responses to the questions were designed based on a 5‐point Likert scale ranging from *strongly disagree* (score 1) to *strongly agree* (score 5). Some questions (4, 6, 7, 8, 9, and 10) were scored reversely. The final score of health information avoidance ranged from 10 to 50; a higher score means more information avoidance. The questions in this section are the same items shown in Table 2.

#### 2.2.2 The eHealth Literacy Questionnaire Section

In this section, the researchers used the Electronic Health Literacy Scale (eHEALS) designed by Norman and Skinner [20]. This tool serves as a standard questionnaire to evaluate perceived eHealth literacy across different age groups, gauging an individual's comprehension of their abilities and knowledge in any health‐related field. Although, in the Norman and Skinner's study, it was used for the age group of 13−21 years [20]. It has been used in various studies for different age groups. For example, Lin et al. examined e‐Health literacy of the groups of patients over 65 years of age using eHEALS [21]. In addition, it offers an overall assessment of a person's skills relevant to eHealth. This 8‐item questionnaire had answers on a 5‐point Likert scale ranging from *strongly disagree* (score 1) to *strongly agree* (score 5). The total score of this tool ranges from 8 to 40, and higher scores indicate a higher level of electronic literacy [20].

In a study conducted by Bazm et al., the validity and reliability of its Persian version were also confirmed [22]. In the present study, the reliability of this tool was also calculated using Cronbach's *α* for 25 people from the community, and the result was 0.93. The questions in this section are the same items shown in Table 3.

**Table 3:** The frequency of patients with MS in terms of eHealth literacy.

eHealth literacy items	Strongly agree F (%)	Agree F (%)	Neither agree nor disagree F (%)	Disagree F (%)	Strongly disagree F (%)
I know what health resources are available on the Internet	10 (9.0)	57 (51.4)	25 (22.5)	16 (14.4)	3 (2.7)
I know where to find helpful health resources on the Internet	9 (8.2)	45 (40.5)	38 (34.2)	14 (12.6)	5 (4.5)
I know how to find helpful health resources on the Internet	8 (7.2)	49 (44.2)	33 (29.7)	15 (13.5)	6 (5.4)
I know how to use the Internet to answer my questions about health	11 (10.0)	53 (47.7)	25 (22.5)	16 (14.4)	6 (5.4)
I know how to use the health information I find on the Internet to help me	12 (10.8)	46 (41.4)	35 (31.6)	12 (10.8)	6 (5.4)
I have the skills I need to evaluate the health resources I find on the Internet	8 (7.2)	47 (42.3)	34 (30.6)	17 (15.4)	5 (4.5)
I can tell high‐quality health resources from low‐quality health resources on the Internet	6 (5.4)	39 (35.1)	39 (35.1)	21 (19.0)	6 (5.4)
I feel confident in using information from the Internet to make health decisions	7 (6.3)	37 (33.3)	38 (34.2)	21 (19.0)	8 (7.2)

### 2.3 Data Analysis

Data analysis was done in two parts: Descriptive and inferential statistics using SPSS version 21. To achieve the descriptive objectives of the study, descriptive statistics indicators, including frequency and percentage, mean, and standard deviation, were used. Pearson's correlation coefficient, *t*‐test, one‐way analysis of variance (ANOVA), and Bonferroni test were used to check the relationship between variables.

### 2.4 Research Limitation

One limitation in this study is related to the participants who were unable to independently complete the questionnaire due to physical limitations, such as the inability to hold a pen or visual impairments. In such instances, researcher #1 assisted them by reading and explaining the questions and entering the answers to the questionnaires with a concerted effort made to avoid any potential bias. A total of 19 patients with MS faced challenges when attempting to respond to the eHealth literacy inquiries, primarily stemming from the lengthening of the questionnaire, their apprehension and anxiety surrounding internet usage, impatience, and visual impairments. Consequently, their cooperation in completing this part of the questionnaire was discontinued. As a result, we analyzed their responses in the health avoidance section, and the analysis for the eHealth literacy section was conducted using a smaller subset of the overall sample size.

Another limitation of this study concerns the sampling method. Because convenience sampling was used instead of random sampling, the sample may not fully represent the target population. This limitation could affect the external validity of the findings, and any generalization to the broader population should be approached with caution.

## 3 Results

### 3.1 Demographic Information

The participants ranged in age from 14 to 71, with an average age of 37. Most of them were female, making up 68.5% of the group, and 70% were married. All participants were literate. Regarding education, 35.4% held a high school diploma, 34.6% had an undergraduate degree, and 6.1% possessed a Master's or PhD. The remaining 23.8% had less than a high school diploma. Regarding English language proficiency, 40% of the participants reported having very low proficiency, 23.8% had low proficiency, 31.5% were at an average level, and 4.6% had high proficiency.

### 3.2 General Health Information Avoidance

Table 1 shows the frequency of the studied subjects according to the level of general health information avoidance by each of their skills in a five‐option Likert scale. In response to the questions about general health information avoidance, most respondents used the “disagree” option, indicating they were against it. The second question was scored inversely, showing that 56 people (43.1%) of the studied people thoroughly agreed to know the information related to their health, even if it made them uncomfortable.

### 3.3 MS Health Information Avoidance

Table 2 illustrates that a significant proportion of patients (45 people, 40.8%) strongly disagreed with items #2 and #3. In other words, they prefer to be aware of novel treatment methods or information about MS. On the contrary, a small minority of individuals (specifically, three individuals) would rather not have information about the progression of the disease or new treatment methods. Most patients strongly agree (57 people, 43.8%) or agree (49 people, 37.7%) that they know their disease status, even if it makes them upset. Most patients (57 people, 43.8%) want to know the information about their MS status. Most patients (69 people, 53.1%) stated that knowing the status of their MS is vital for them. Seventy‐nine people (60.8%) know that it is important for them to know the novel control and treatment methods for MS disease, and they strongly agree that they should know the information about the progression of their MS disease immediately after the MRI.

### 3.4 eHealth Literacy in Patients With MS

Regarding the components of eHEALS, Table 3 shows the frequency and percentage of responses related to each component. Most participants reported their answers on a 5‐point Likert scale at the “Agree” level in all components of eHealth literacy. The components of “enough confidence in using Internet information for medical and health decisions” and “ability to distinguish high‐quality and reliable health information sources from low‐quality and unreliable Internet sources” have obtained lower scores than the other components.

### 3.5 Correlation Between eHealth Literacy, Health Information Avoidance, and Demographic Variables in Patients With MS

Table 4 shows that the average score of the respondents to the questions on general health information avoidance was 9.06 out of 20. The average score of the respondents to the questions about MS health information avoidance was 20.52 out of 50. The respondents' average score on the eHealth literacy questions was 26.28 out of 40.

**Table 4:** Descriptive statistics related to health information avoidance (general and MS) and eHealth literacy.

	Score range	Number	Minimum	Maximum	Mean	Standard deviation
General health information avoidance	4−20	130	4	17	9.06	3.00
MS health information avoidance	5−10	130	10	40	20.52	6.19
eHealth literacy	8−40	111	8	40	26.28	5.95

The values in Table 5 are Pearson's correlation coefficients (*r*), and *p*‐values (*p*) in the parenthesis. Correlation analysis showed that general health information avoidance exhibited a moderately strong association with MS health information avoidance (*r* = 0.573, *p* < 0.001). However, eHealth literacy showed no significant relationship with general health information and MS health information avoidance (*p* ≥ 0.05) (Table 5).

**Table 5:** The matrix of the relationship between eHealth literacy and information avoidance in patients with MS; *r* (*p*).

	eHealth literacy	General health information avoidance	MS health information avoidance
eHealth literacy		0.041 (0.64)	−0.007 (0.94)
General health information avoidance	0.041 (0.64)		0.573 (≤ 0.001)^a^
MS health information avoidance	−0.007 (0.94)	0.573 ( ≤ 0.001)^a^	

^a^ Pearson's correlation was significant at the 0.05 level (two‐tailed); (*p* < 0.05).

The Student *t*‐test, ANOVA, or Pearson's correlation coefficient tests showed that there was no significant correlation between any of the demographic variables (age, marital status, educational degree, the level of English language proficiency) and avoidance of general and MS health information (*p* ≥ 0.05).

Furthermore, no significant correlation was identified between age, marital status, educational level, and e‐health literacy. The only significant correlation was found between the level of English language proficiency and eHealth literacy, examined using one‐way ANOVA showed that the levels of health literacy in patients with various levels of English language proficiency is significantly difference (*p* = 0.006). The Bonferroni test showed that the level of health literacy in the patients with the higher levels of English language proficiency (average and high levels) was higher than in other groups (low and very low levels) (*p* < 0.05).

## 4 Discussion

Ignoring health information can endanger health, transmit and spread disease, and, in some cases, impose costs on patients, families, and societies. Therefore, avoiding health information has irreparable consequences and complications for the individual and society. E‐health literacy is also a crucial skill for retrieving and using health information. This study investigated health information avoidance and eHealth literacy in patients with MS, as well as their correlation with each other.

The results indicate that patients with MS, on average, scored 9.06 out of 20 on the avoidance of general health information scale. Meanwhile, the score for information avoidance specific to MS averaged 20.52 out of 50, below the median range. Notably, a lower score on the information avoidance scale is deemed more favorable. In this regard, the research of Schumacher et al. showed that the average score of information avoidance related to weight was 23.2 and less than half in many people [17], almost at the same level as the present study findings. Howell and Shepperd's research on information avoidance presented the average avoidance levels for three distinct groups. The group avoiding information about attractiveness had an average avoidance of 4.05. In contrast, the group avoiding health‐related information averaged 2.44, while the group that avoided information concerning partner betrayal averaged 1.87 [8]. Their findings closely align with recent research regarding general health information avoidance. The study conducted by Naderbeigi and Isfandyari‐Moghaddam reported that students from Hamedan University of Medical Sciences, on average, rated their health information avoidance at 2.21 out of 5 [16], nearly equivalent to the findings of the present study.

The average eHealth literacy score in the present study from all patients who answered the eHealth literacy questions is 26.28 out of 40 scores, and above the average. Notably, a higher score indicates a better state of eHealth literacy. Soleimaninejad et al. reported the average eHealth literacy in 160 caregivers of elderly with high blood pressure and type 2 diabetes to be 26.16, which aligns with this study [23]. In another study by Rasouli et al., the average eHealth literacy score in patients referred to a military hospital was 25.3, slightly lower than the present study [24]. In addition, Zahedi et al. reported the average health literacy in hemodialysis patients as 14.88, lower than the average of the current study [25]. In some previous studies, the average eHealth literacy of patients has been reported to be higher than in the present study. For example, Isazadeh et al. reported the average eHealth literacy score of patients referred to a selected military hospital in Tehran, Iran, to be 29.28, higher than this study [26]. The findings of Valizadeh‐Haghi and Rahmatizadeh indicated that the average eHealth literacy of patients with dental diseases is 30.55 [9]. In the study of the relationship between eHealth literacy and breast cancer literacy among Saudi women, Almoajel et al. reported the average eHealth literacy of the participants to be 28.79 and breast cancer literacy to be 23.44 [27]. Ghaedi et al. also reported the eHealth literacy score of students of Shahid Beheshti University of Medical Sciences as 29.29 out of 40 [28], which is higher than the eHealth literacy score of patients with MS, as well as the patients examined in some previous studies [23, 27]. The variations observed might be attributed to the disparities within the research communities, highlighting that strategies for improving health literacy must be tailored to each specific community. This customization should be based on careful assessments conducted within the relevant field. Furthermore, it is often observed that students tend to score higher on eHealth literacy than patients. This could be attributed to their access to education focused on eHealth literacy, such as courses teaching how to search for and evaluate information sources.

The results show no significant correlation between eHealth literacy and health information avoidance with the demographic variables, except for the English language proficiency, which correlates significantly with eHealth literacy. These findings are consistent with those of some previous studies, indicating no significant correlation between eHealth literacy or health information avoidance and demographic variables such as age and sex [29, 30, 31] [17, 19]. However, differences exist between the findings of the present study and some previous studies, showing that patients' age significantly affects their eHealth literacy level [9, 27]. The present study results also show that eHealth literacy has no significant relationship with general and MS health information avoidance. Seemingly, patients with MS have a strong motivation and desire to obtain information. Hence, their information avoidance is low and is unaffected by other variables. These findings do not align with Siebenhaar et al.'s findings, indicating that people with a higher level of eHealth literacy have experienced less worry and anxiety during COVID‐19 and, as a result, have less information avoidance [18]. St. Jean et al.'s research revealed that low information literacy significantly influences information avoidance. Their study found that individuals with limited health‐related information literacy primarily avoided information, contradicting the current study [4]. Given the limited number of studies and the varying results, further exploring this context through additional research is recommended. Additionally, as noted by Orom et al. [32], it is essential to implement interventions that focus on tailored messaging for individuals aimed at enhancing eHealth literacy and minimizing the tendency to avoid health information.

## 5 Conclusion

The present study scrutinized how patients with MS approach health information, particularly their tendency to avoid it, and how this correlates with their eHealth literacy. The obtained results revealed that patients with MS exhibit a high level of interest in understanding their condition, including various disease aspects. Interestingly, their avoidance of information was found to be below average. Recent studies have shown a higher level of information avoidance regarding COVID‐19 and obesity, suggesting that the extent to which people avoid general health information or details related to specific diseases can vary greatly. This variation often depends on several factors, most notably the type and nature of the disease.

Although no substantial correlation was discovered between eHealth literacy and the tendency to avoid health information, the strong desire of patients with MS to acquire health information and their low avoidance scores suggest the necessity for strategic planning. This planning should focus on preparing and sharing the relevant information required by these patients. Besides, planning and policy‐making are needed to enhance the eHealth literacy of patients with MS. Based on the scores achieved in eHealth literacy and the health information (general and MS), it is recommended that workshops and training sessions be held. These can effectively improve eHealth literacy and decrease instances of information avoidance.

## Author Contributions

**Leila Mobasher:** conceptualization, methodology, software, data curation, investigation, validation, formal analysis, resources, visualization, writing – original draft, writing – review and editing. **Maryam Shekofteh:** conceptualization, methodology, investigation, supervision, funding acquisition, visualization, project administration, resources, writing – original draft writing – review and editing. **Maryam Kazerani:** conceptualization, methodology, investigation, resources, writing – review and editing. **Sara Jambarsang:** methodology, software, validation, formal analysis, visualization, writing – review and editing. **Maryam Poursadeghfard:** methodology, data curation, investigation, writing – review and editing. All authors have read and approved the final version of the manuscript.

## Ethics Statement

This study was approved by the Iran National Committee for Ethics in Biomedical Research. The code of ethics is IR.SBMU.RETECH.REC.1402.483. This project is based on an MS thesis with the code of IR.SBMU.RETECH.REC.1401.663. All participants provided written informed consent for their participation in the study. In fact, the inclusion criterion for the study was the patients' consent to participate in the study. Since some participants were under 18, we also obtained written informed consent from their parents.

## Consent

All methods were carried out in accordance with relevant guidelines and regulations. Identified images or other personal or clinical details are not presented in this manuscript.

## Conflicts of Interest

The authors declare no conflicts of interest.

## Transparency Statement

The lead author, Maryam Shekofteh, affirms that this manuscript is an honest, accurate, and transparent account of the study being reported; that no important aspects of the study have been omitted; and that any discrepancies from the study as planned (and, if relevant, registered) have been explained.

## Data Availability

The data used to support the findings of this study were supplied by corresponding author and will be available upon reasonable request.
